# Analysis of porphyrins from serum and the benefit of inert biphenyl columns for liquid chromatography separation

**DOI:** 10.1371/journal.pone.0357115

**Published:** 2026-09-15

**Authors:** Moritz Unnerstall, Manfred Fobker, Patrick Christian Opitz, Lukas Helmer, Eric Suero Molina, Simone König

**Affiliations:** 1 Service Unit Proteomics, Medical Faculty, University of Münster, Münster, Germany; 2 Clinical Laboratory, University Clinic Münster, Münster, Germany; 3 Department of Neurosurgery, University Clinic Münster, Münster, Germany; The Chinese University of Hong Kong, HONG KONG

## Abstract

Reversed-phase liquid chromatography (LC) is a major separation technique for porphyrins. We coupled C18-based LC with mass spectrometry (MS) for the detection of protoporphyrin IX (PPIX) in the context of neurosurgery, where it is used as a fluorescence marker for tumor visualisation. Here, we demonstrate advantages of the replacement of the C18 with an inert biphenyl (iBP) column, which improved separation resolution and increased detection sensitivity in LC-MS of six porphyrins (uroporphyrin I, hepta-, hexa-, and pentacarboxyporphyrin I, coproporphyrins I and III) associated with the heme biosynthesis pathway. They are of potential interest as intermediates and side products in our research on 5-aminolevulinic acid-induced fluorescence-guided neurosurgery. Porphyrins consist of conjugated π-systems which interact with aromatic stationary phases such as BP by taking advantage of π-π stacking interactions. Carryover observed with PPIX on endcapped C18 stationary phase was reduced. This improvement was due to the reduction of non-specific adsorption on metallic surfaces from the LC instrument by using inert column hardware combined with the BP stationary phase. Passivation by chemical vapor deposition was shown to reduce non-specific adsorption in the LC system better than methods using mobile phase additives. PEEK tubing in conjunction with the iBP-column provided the best results and shortened run time. Furthermore, we present a Python-based software tool for automated data processing and analyte quantification. This work contributes to our ongoing developments of porphyrin LC-MS in blood and tissue samples from glioma patients.

## Introduction

Reversed-phase liquid chromatography (RP-LC) is a major separation technique in biomolecular analysis [1] and the favoured mode for small molecule pharmaceutical analysis [2]. It has also been used for chromatography of porphyrins [3–10]. We coupled C18-based LC with mass spectrometry (MS) for the detection of protoporphyrin IX (PPIX) in serum for monitoring of brain tumour progression via liquid biopsy [7,8] and in malignant glioma tissue [11]. Our method was, however, still in need of improvement, because we struggled with carryover when PPIX was measured at higher concentrations, i.e., pmol amounts on-column.

A major step forward was the use of an inert biphenyl (iBP) column [12]. Regular BP columns have been on the market for some time and were used, for instance, for the detection of steroids and drugs [13–16]. For steroid analysis, it was additionally shown that replacing LC conventional hardware by passivated hardware, combined with an inert column packed with BP stationary phase, considerably reduced peak width and increased sensitivity [12]. This improvement was due to the reduction of non-specific adsorption on metallic surfaces from the LC instrument and the column [17], a phenomenon, which has been reported to limit, in particular, the analysis of phosphorylated biomolecules [18,19]. The use of inert column hardware (tubing, frits, frit retainers) generated by chemical vapor deposition (CVD) [20] was shown to mitigate non-specific adsorption [12,21]. Among other methods for the passivation of LC systems like mobile phase additives (e.g., medronic acid) [17], CVD appears to be the most robust method. It should also be noted that replacement of metal parts with fused silica material does not entirely resolve the problem, because of the capacity of silanol moieties on silica surfaces to adsorb; endcapping of the free silanol groups was introduced to minimize this effect [19].

**Fig 1:**
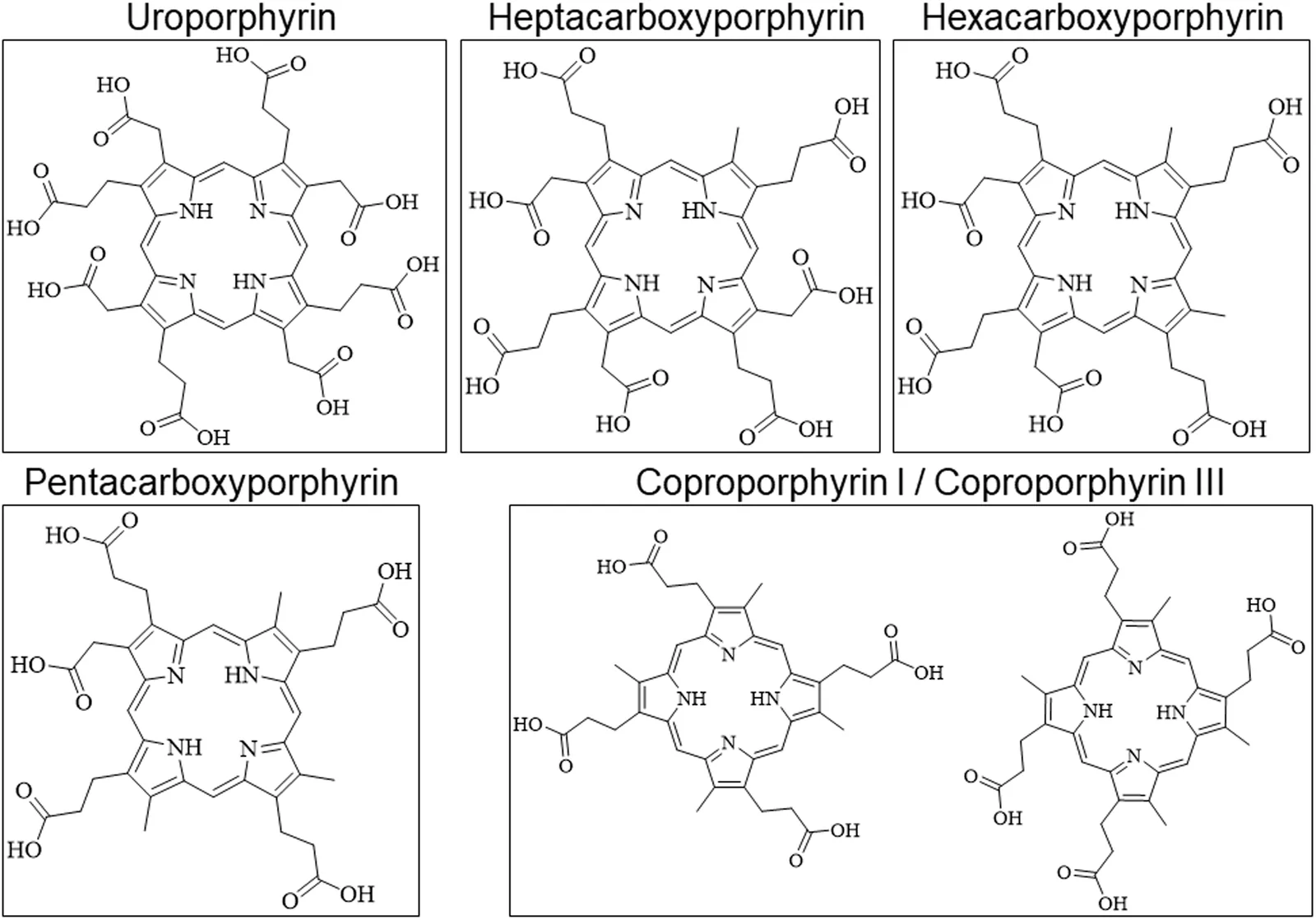
Structures of the porphyrins investigated in this study. Uroporphyrin I (Uro), heptacarboxyporphyrin I (Hepta), hexacarboxyporphyrin I (Hexa), pentacarboxyporphyrin I (Penta), and coproporphyrins (Copros) I and III.

Metal-analyte interactions have been observed for several classes of compounds having acidic or charged moieties [12,21], but were not discussed for porphyrins so far. However, a basic function of these molecules is metal-binding (e.g., iron in heme) [22,23] so that it can be expected that they interact with metal surfaces. Moreover, porphyrins consist of conjugated π-systems (Fig 1) which interact with aromatic stationary phases such as BP by taking advantage of π-π stacking interactions [24].

Here, we present results for the separation of six porphyrins associated with the heme biosynthesis pathway (Fig 1). They are of potential interest as intermediates and side products in our research on 5-aminolevulinic acid-induced fluorescence-guided neurosurgery [7,8,11,25,26]. We used both endcapped C18 and iBP stationary phase LC columns and discuss their performance and limitations. Moreover, we present a software tool for automated data processing and analyte quantification.

## Materials and methods

The study is based on the ClinTest standard from Recipe (Munich, Germany) containing uroporphyrin I (Uro), heptacarboxyporphyrin I (Hepta), hexacarboxyporphyrin I (Hexa), pentacarboxyporphyrin I (Penta), and coproporphyrins (Copros) I and III (Fig 1).

All experiments were performed in accordance with the declaration of Helsinki and with approval of the local ethics committee of the University of Münster and the Ärztekammer Westfalen-Lippe (2017–169-f-S).

Chemicals and solvents were obtained from Sigma-Aldrich unless otherwise noted. Acetonitrile (ACN) HiPerSolv Chromanorm was purchased from VWR, Copro I, formic acid (FA) and acetic acid glacial from Merck. Methanol (MeOH) ULC-MS-CC/SFC came from Biosolv and later from Honeywell / Riedel de Haën along with the LC-MS Chromasolv water, and Copro III was from Frontier Scientific. Mesoporphyrin (MPIX) was dissolved in dimethylsulfoxide (DMSO) at 10 pmol/l as stock solution and used for process control.

### Anion-exchange solid-phase extraction

Purification of the ClinTest standard was performed using anion-exchange solid-phase extraction (aeSPE; OASIS^®^ MAX cartridge, 3 ml, 60 mg, Waters Corp.) with a 12 channel vacuum station (Millipore). For method development, 20 µl ClinTest standard, 80 µl water, 400 µl of diluted MPIX solution (0.05 pmol/µl) and 1 ml ACN were vortexed and loaded onto the cartridge. It was washed with 2 ml of aqueous 2.5% NH_4_OH followed by 2 ml of MeOH. The analytes were eluted with 2 ml of 20% FA in ACN. After each washing step and the elution, a slight vacuum (−2.5 kPa) was applied to the cartridge for 30 s. The eluate was dried in a vacuum centrifuge (Concentrator 5301, Eppendorf) at 30 °C. Then, the sample was reconstituted with 40 µl DMSO (for later experiments, DMSO with 20% aqueous 0.1% FA was used – see Results Section *Injection solvent*) and filtered through a centrifugal filter unit (Nanosep® 0.2 µm wwPTFE, Pall Life Sciences) for subsequent analysis.

### LC-MS of porphyrins

Analysis of the purified ClinTest porphyrins was performed with LCMS-8050 instrument (Shimadzu, Kyoto, Japan). For C18 LC, parameters were: flow rate 0.3 ml/min, injection volume 10 µl, column temperature 35 °C, solvent A: 0.1% FA in water, solvent B: ACN containing 0.1% FA; for 25 min runs: gradient 0–20 min 5–65% B, 20.5–22.5 min 95% B; using tread vials amber glass 1.5 ml 32 x 11.6 mm (Neochrom) with silanized inserts (15 mm tip, 0.2 ml, Machery Nagel GmbH & Co. Kg) and Verex Caps (preassembled w/ Bonded-In PTFE/Silicone septa, Phenomenex). Buffered solvent using 10 mM ammonium acetate in water (solvent A, pH 5.6) versus ACN as solvent B was tested for completeness sake as recommended [27] (see Results Section *LC-MS method*). The autosampler temperature was adjusted to 23 °C accommodating the melting point of DMSO at 18.5 °C. For iBP LC, according to the recommendations of the manufacturer, MeOH and water, each containing 0.1% FA, were selected as mobile phases. LC columns were Poroshell 120 EC-C18 (Agilent, Waldbronn, Germany) and Raptor Biphenyl (Restek, Bellefonte, Pennsylvania, USA), both 100 x 2.1 mm, particle size 2.7 µm. The column temperature was 35 and 40 °C for C18 and iBP, respectively. The elution sequence of the Copros was determined using the individual reference compounds.

MS was conducted in positive electrospray mode (nebulizer and drying gas flow 3 l/min, interface temperature 350 °C, heating gas flow 17 l/min, product ion scan range *m/z* 400−800, collision energy −54 V except for Copros I/III (−48 V)). Compound-specific target ions for multi-reaction monitoring (MRM) are shown in Table 1. Porphyrins primarily fragment at the side chains as discussed before [8,11,28]. Data were analyzed using instrument-specific software LabSolutions v.5.89 (Shimadzu). The areas of the quantifier ions were evaluated with Skyline (v.23.1.0.268, MacCoss Lab Software) and the mean signals of replicates were compared by calculating the standard deviation with OriginLab 2024 v.10.1.0.170 (OriginLab Corp., Northampton, USA).

**Table 1:** Compound-specific analyte target ions for MS analysis of the ClinTest standard. Qualifier and quantifier ions for quantification analysis. For exemplary fragmentation spectrum, see S1 File.

Analyte	MH^+^	*m/z* Quantifier Ion	*m/z* Qualifier Ions
Uro	831.25	727.25	785.25; 767.25
Hepta	787.25	683.25	728.25; 669.25
Hexa	743.25	639.25	684.25; 625.25
Penta	699.25	595.25	640.25; 581.25
Copros I/III	655.25	596.25	537.25; 523.25

### Validation of the LC-MS method

The MRM method was tested with the C18-column in human urine as biological matrix using the Chromsystems standard (Gräfeling, Germany), which also contained the same analyte porphyrins (Table 2). In order to purify the porphyrins from the formulation and reduce sample complexity, above aeSPE method was applied. To that end, lyophilized standard was dissolved in 2 ml water of which 100 µl aliquots were subsequently used. Prior to the aeSPE procedure, 400 µl of MPIX solution (0.05 pmol/l) was added to each aliquot as quality control of the aeSPE procedure followed by 1 ml ACN for protein precipitation with 1 h shaking, respectively, centrifugation and aeSPE. Stable MPIX detection ensured confidence in sample preparation. Different injection volumes were used for LC-MS in order to test linearity (Table 2).

**Table 2:** Porphyrin concentrations for analysis. Values in Chromsystems urine standard after aeSPE and approximate injected amounts on column (pmol).

Analyte	Uro	Hepta	Hexa	Penta	Copro I	Copro III
**Concentration / (pmol/µl)**	0.200	0.0343	0.0363	0.0355	0.135	0.286
**Injection volume / µl**						
1	1	0.17	0.18	0.18	0.67	1.43
3	3	0.51	0.54	0.53	2.02	4.29
5	5	0.86	0.91	0.89	3.37	7.15
6	6	1.03	1.09	1.06	4.05	8.58
8	8	1.37	1.45	1.42	5.40	11.44

### Validation of aeSPE

The reproducibility of the extraction procedure was evaluated in repeated aeSPE experiments using the ClinTest standard. The purification was performed independently three times with sample containing 0.1 pmol/µl of each porphyrin. Triplicates were run with LC-MS (C18 column).

### Injection solvent

We aimed to reduce the amount of DMSO injected into the LC system by replacing it with varying amounts of LC solvent A or B using one batch of aeSPE-purified ClinTest standard (200 µl). Six 300 µl aliquots were prepared from the eluate and dried. They were subsequently reconstituted using varying compositions of DMSO, ACN with 0.1% FA, and aqueous 0.1% FA. Every sample was reconstituted with 40 µl of solvent and triplicate injections were measured with LC-MS (C18 column).

### Matrix effects in iBP analysis

For testing of matrix effects, 500 µl serum from a healthy volunteer was spiked with 40 µl of the ClinTest standard and compared with an unspiked sample. Aqueous MPIX (2 ml, 0.05 pmol/µl) was added and the solution was shaken at 1000 rpm for 1 h. Then, 5 ml ACN were added with subsequent 1 h shaking for protein precipitation. The supernatants were purified via aeSPE. Additionally, a blank sample was prepared without serum using 500 µl H_2_O instead. The entire process was repeated for 100 µl serum as starting material, adjusting volumes to 400 µl MPIX solution and 1 ml of ACN.

### Standard addition procedure for porphyrin quantification

Serum of a healthy volunteer (100 µl per experiment) was spiked with different levels of ClinTest standard (5, 10, 20, 40, 50 µl) and aeSPE was conducted followed by LC-MS (iBP column). For data analysis, a software tool was developed using Python 3.14 and Visual Studio Code (download September 2025).

### Liquid-liquid extraction of serum

We spiked 100 µl serum from a healthy donor with 20 µl ClinTest standard and diluted it with 200 µl water followed by the addition of 20 µl 18% HCl and vortexing. Subsequently, 200 ml of organic solvent (ethyl acetate, chloroform, dichloromethane, toluene, respectively) were added and vortexed. Phase separation was induced by centrifugation at 15000 rpm for 5 min. The organic phase was collected, dried and reconstituted in 40 µl DMSO / aqueous 0.1% FA (80/20 v/v) for LC-MS (iBP column).

### Evaluation of carryover

The extent of residual PPIX in the LC system (HP1100, Agilent, Waldbronn, Germany; coupled to Esquire 3000 ion trap, Bruker Corp., Bremen, Germany) was studied for the same LC-columns and solvent systems as described above using metal or PEEK tubing, respectively, and optional system passivation by medronic acid as recommended by the manufacturer (Restek, Bellefonte, PA, USA). Method parameters for LC were: flow rate 0.3 ml/min, gradient for a 15 min run: solvent B 10–100% in 0.5 min, 6 min hold for 100%, and for MS ESI (+): nebulizer pressure 2.07 bar, dry gas flow 7 l/min, dry temperature 300 °C, scan ranges *m*/*z* 250–800 (angiotensin II, Ang II) and *m*/*z* 400–600 (PPIX), MS/MS Ang II at *m*/*z* 523.8 ([M + 2H]^2+^) and amplitude 0.56 V, MS/MS PPIX at *m*/*z* 563.3 (MH^+^) and 1.10 V (quantifier *m*/*z* 504.3, qualifiers *m*/*z* 445.3 / 489.3) [7]. Data analysis was performed using SkyLine software (Version 25.1) and Bruker Daltonics DataAnalysis (v.3.0, Bruker Corp., Bremen, Germany). Analyte concentrations were: PPIX 0.001, 1, 5 pmol/µl and Ang II 1 pmol/µl in DMSO in 10 µl injections.

## Results and discussion

### Porphyrin reference substances

Unfortunately, no porphyrin standard was commercially available, which could be used for MS experiments without prior treatment. The ClinTest product provided six of the porphyrins of interest, but supposedly contained considerable amounts of polyethylene glycol causing severe suppression effects [29]. Uro and Hepta could not be detected at all without prior purification. Fortunately, our aeSPE procedure, which we had established for porphyrin extraction from serum earlier [7], was also suitable for its purification, but, of course, affected the given porphyrin concentrations.

### LC-MS method

We used the purified standard to set up an LC-MRM method with the C18-column and the FA/water/ACN solvent system (for exemplary spectral data, see S1 File), which we subsequently validated using a different commercial porphyrin standard containing human urine. This material was also purified by aeSPE and was analyzed with correlation coefficients for all analytes exceeding R^2^ = 0.990 proving the reliability of method. The use of silanized compared to non-silanized inserts for LC-vials provided not much of an advantage; only about 1.1-fold more intensity was obtained for Uro, Hepta and Hexa and no change for the other porphyrins.

For completeness sake, we also tested buffered solvent A (pH 5.6) as recommended by Shimadzu for porphyrin measurement in urine with fluorescence detection [27]. However, MS intensities were more than 2-fold (Copros) and up to 18-fold (Uro) reduced with the values for the other analytes ranging in-between.

### Reproducibility of aeSPE

The reliability of the aeSPE method was evaluated by repeated purification of the ClinTest standard and comparison of the results. The standard deviation of the detected amounts for the analytes was between 5.6% (Copro I) and 8.9% (Hepta) (S1 File) and acceptable for bioanalytical method validation. The results indicated consistent extraction efficiencies across three replicates and were within established criteria [30] of SPE -based sample preparation workflows for human sera. It should be noted that Uro, being the most hydrophilic of the investigated porphyrins, behaved somewhat sensitive to subjective factors such as the choice of vacuum regime when different persons performed the aeSPE procedure.

### Injection solvent

In previous studies [7,11], LC-MS analysis was carried out using DMSO as a porphyrin solvation and injection solvent, but it has substantial drawbacks. Larger amounts of DMSO can lead to swelling of PEEK capillaries resulting in reduced operational life spans of tubing material. It can also cause backpressure in the LC system due to its high viscosity. Another significant disadvantage of DMSO is its freezing point of 18.5 °C, which necessitates an appropriate temperature setting in the autosampler. This limitation negatively impacts on the stability of heat sensitive analytes and prohibits automatic measurements of large sample numbers. Thus, we tested mixtures of DMSO with LC solvents to possibly reduce its amount for analysis. ACN + 0.1% FA and H_2_O + 0.1% FA used without DMSO reduced signal intensities significantly (S1 File) and the best results were achieved with mixtures containing at least some DMSO. Since the combination DMSO / aqueous 0.1% FA was equally successful as ACN-containing formulations, the mixture was finetuned by repeating the experimental setup for different ratios of this mixture (S1 File). The highest signal intensities could be achieved for 80/20 (v/v) DMSO / aqueous 0.1% FA. Thus, this solvent composition was subsequently utilized for injection as a way to mitigate the adverse effects of DMSO. Notable effects on the peak shape caused by the injection solvent were not observed.

### LC with iBP stationary phase

**Fig 2:**
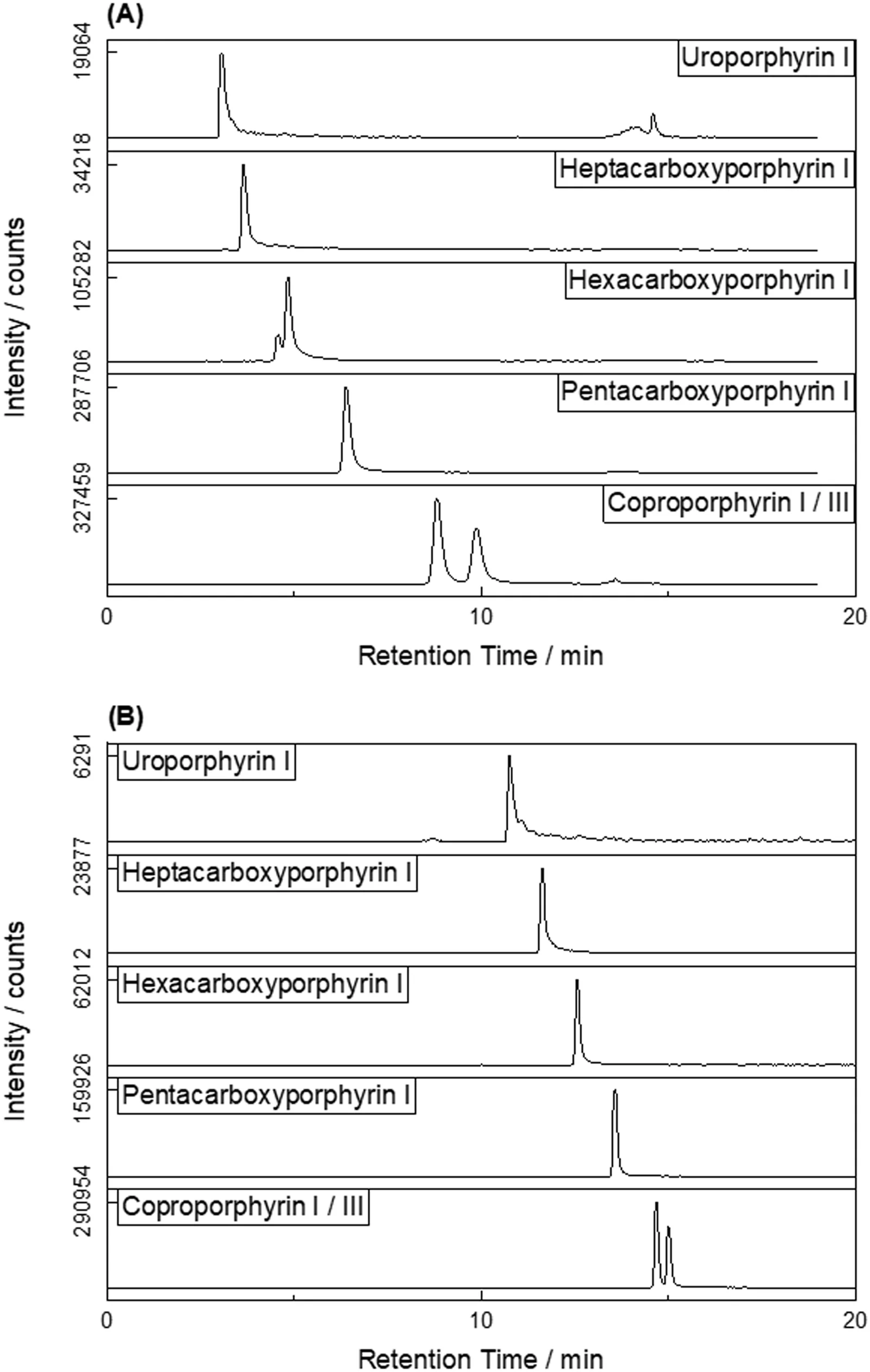
Comparison of the column performance. (A) iBP column and (B) C18 column for the chromatographic separation of the six porphyrins (summed intensity of all ions selected for MRM of the individual compounds).

We tested the performance of the LC-MS method for the ClinTest porphyrins using the iBP column. High chromatographic resolution was achieved in comparison to C18 separation in shorter 19 min runs by a steep gradient increase over 0.5 min from 30% B to 65% B with a subsequent 11-minute isocratic hold (Fig 2). Copros I/III were baseline-separated with Copro I eluting before Copro III. The Hexa peak showed partial separation of two Hexa isomers contained in the ClinTest standard (Hexa I ab and Hexa I ac, as specified by the manufacturer), but since they were not individually available for reference, further separation was not attempted. Collision energies were finetuned to improve the signals of the qualifier ions (Uro −50 V, Hepta −65 V, Hexa −54 V, Penta −52 V, Copro I/III −48 V) and the interface temperature was decreased to 325 °C. Signal intensities were equal (Hepta) or improved 2- (Uro, Copros) to 3-fold (Hexa, Penta) compared to the C18-column.

### Matrix effects

We further studied, if human serum as the matrix of interest had any influence on analysis performance. While this concerned LC-MS in general, we worked with the iBP column for its better performance. When 500 µl serum was purified and analyzed, the signal of Uro was completely suppressed, all other analytes could be detected, but at reduced intensity (S1 File). When the process was started with 100 µl serum, however, the influence of residual matrix was eliminated, and all porphyrins were detected at about the same intensities as for the experiment starting with spiked water (S1 File). In unspiked serum, endogenous Copros were noted as expected [31].

### Porphyrin quantification and data processing

No dedicated porphyrin standard for MS measurements was available in this study, nor were isotope standards of the porphyrins of the heme biosynthesis pathway. Since the ClinTest standard required aeSPE before use, quantification accuracy was limited, because of possible sample loss during the procedure. MPIX was also not well suited as an internal reference, because it tended to generate carryover. Thus, we used the standard addition procedure. All analytes showed a linear response with coefficients of determination R^2^ > 0.990 (S1 File). No significant carryover effects were detected.

The calibration curves were used to estimate the limits of detection (LOD) and quantification (LOQ). However, serum might contain endogenous porphyrins so that LOD and LOQ could only be approximations. Considering the EMA validation guidelines [32], LOD and LOQ were calculated with the standard deviation of the response σ and the slope of the linear calibration *m* (Table 3)*.*

**Table 3:** Limit of detection. LOD and LOQ of the porphyrins (fmol/µl).

Analyte	LOD	LOQ
Uro	64	193
Hepta	43	131
Hexa	38	114
Penta	14	41
Copro I	24	73
Copro III	21	64


$$\begin{array}{c} LOD=\frac{\text{3}.\text{3}\sigma }{m}\text{ and }LOQ=\frac{\text{1}\text{0}\sigma }{m} \end{array}$$

The standard addition procedure compensated for matrix effects, but was labor-intensive including time-consuming data processing as each sample required its own dedicated calibration curve and mathematical evaluation [33]. Thus, a dedicated Python code was developed with the assistance from artificial intelligence tools (OpenAI ChatGPT, Microsoft Copilot) to reduce manual processing of LC-MS data. The objective was an algorithm that enabled scalability of porphyrin analysis using the standard addition procedure in a clinical setting with large data sets or many analytes. Python, an open-source programming language, allowed partial automatization of calculation, statistical analysis and visualization of the analysis results. Input was a csv-file containing sample and analyte name, spike level, and measured peak area (see S1 File for information on the Python tool). The output consisted of an Excel table reporting the calculated sample concentration in unspiked samples, measurement uncertainty, and the regression equation with the respective R^2^. Additionally, png-files were generated for visual examination of the regression curves. The images contained, beside the plot and equation of the regression, the mean signal of each data point with the standard deviation of the replicates as error bars and a 95% confidence band for the mean fitted values (S1 File). The x-intercept was depicted with an error bar representing the combined uncertainty. The code script and further information on the folder structure is available in the Supplement (see S1 File, S2 File, S3 File). All calculations were independently verified by manual calculation of exemplary values and randomly selected real sample results using Origin.

### Liquid-liquid extraction of serum

The aeSPE procedure allowed both efficient purification of the ClinTest standard and porphyrin extraction from serum for LC-MS. However, it was too labor-intensive and costly for routine application in a clinical laboratory. Furthermore, the sample throughput was limited by the number of ports of the vacuum manifold used for SPE. Additionally, the need for the standard addition procedure increased the number of experiments. For the analysis of acidic compounds in biological matrices, liquid-liquid extractions (LLE) are commonly used, because of their high extraction efficiencies and simple workflow. Acids are protonated by the addition of an acid and then extracted with an organic solvent, which is immiscible with the other phase [34,35]. When ethyl acetate was used for porphyrin extraction from water, all analytes were successfully detected albeit at much lower intensity (15–18%) than for aeSPE (S1 File). However, when LLE was performed from serum, significant signal suppression was observed and only peaks for Copros I/IIII and Penta could be found at ~2% of the intensity achieved with aeSPE. We further tested chloroform, dichloromethane and toluene with little success and further difficulties. Chloroform and dichloromethane had a higher density than water so that the organic phase had to be harvested from the bottom of the sample vial through a layer of precipitated material dividing aqueous and organic phase, hindering solvent removal. Even worse, no significant analyte peaks were detected when using these solvents. Thus, LLE is not advisable for general porphyrin extraction from serum in this concentration range, but could prove useful for Copro analysis or possibly other matrices such as urine. On a side note, we had tested an even simpler method, which only acidified plasma and used the supernatant for LC-MS [36] before with similar results. While the method generated comparable data to aeSPE for the Copros, the detection of PPIX and MPIX was seriously impaired [31].

### Evaluation of carryover

In our earlier studies, we had noted considerable carryover for PPIX and MPIX [7]. In order to determine its extent and possibly reduce it by using the iBP column, we performed a series of comparative experiments using both metal and PEEK tubing with the C18 and the iBP column, respectively. Carryover was highest in the C18-metal setting requiring eight blank runs following a 10 pmol PPIX injection for the removal of residual signal and lowest for iBP-PEEK needing only two. For a 10 pmol PPIX injection in the C18-metal LC system, about 40 fmol were temporarily adsorbed; for 50 pmol, it were almost 200 fmol. The use of iBP column and PEEK tubing reduced these values to ~10 and 80 fmol, respectively. Passivation of the LC system with medronic acid divided the number of blanks by two when 50 pmol PPIX were injected in the iBP-metal setting, but was, of course, only a temporary fix generating potential variation in quantification of serial experiments [17]. We noted about 3% loss of PPIX intensity over the course of six 50 pmol-injections followed by blanks. By co-running the peptide Ang II, which did not show any signs of unspecific adsorption in the LC system, we confirmed the sensitivity of porphyrin structures for this effect.

## Conclusion

This work adds to our ongoing improvements of LC-MS for the detection of porphyrins and, in particular PPIX, in the context of our collaborative neurosurgical research project [7,11,25]. We compared C18- and iBP-based stationary phases for LC-MS of porphyrins related to the heme biosynthesis pathway. PEEK tubing in conjunction with an iBP-column provided the best results reducing PPIX carryover, shortening run time and improving analyte resolution at similar or higher detection intensity. Porphyrins are prone to unspecific adsorption, but the use of passivated hardware can alleviate the effect.

The lack of suitable porphyrin MS reference substances necessitated the use of a standard addition protocol for porphyrin quantification, for which we developed a Python-based software tool to reduce analysis time. Automated procedures for large sample numbers requiring long run times are still not advisable, because of the necessary temperature in the autosampler close to room temperature and the corresponding subtle concentration changes resulting from evaporation in the LC-vial over time.

aeSPE was the preferred sample preparation method for porphyrins in general for serum compared to LLE. In case that only certain porphyrins such as the Copros are of interest, LLE could be reconsidered as well as the use of buffered solvents in C18-LC-MS.

For our work on porphyrins of the heme biosynthesis pathway [7], the iBP-based protocol needs to be further refined to accommodate the low analyte concentrations in human sera. Preliminary experiments detected only Copros at concentrations <35 pmol/ml in serum [31,37]. For further investigations, calibration ranges need to be adjusted to these lower levels to improve quantification. Moreover, the analytical method has to be adapted to accommodate larger serum volumes in order to increase the chances of the detecting lower abundant porphyrins.

## Supporting information

S1 File Supplementary information. Additional data and instructions for the Python tool.
(PDF)

S2 File Python script. Software file to be used in Python.
(PY)

S3 File Environmental file for the R script. The file (type get-pip) needs to be additionally loaded to run the S2 File in R.
(PY)
