# Supplementary material for: Analysis of porphyrins from serum and the benefit of inert biphenyl columns for liquid chromatography separation
Source: PLoS One. 2026 Sep 15;21(9):e0357115. doi: 10.1371/journal.pone.0357115 (PMC13577369; doi:10.1371/journal.pone.0357115)
Supplement: Supplementary file 1 [file pone.0357115.s001.pdf]

Analysis of porphyrins in serum and the benefit of inert biphenyl columns for liquid chromatography separation

Moritz Unnerstall[1], Manfred Fobker[2], Patrick Christian Opitz [2], Lukas Helmer[1,3], Eric Suero Molina[3], Simone König[1]*
[1]Service Unit Proteomics, Medical Faculty, University of Münster, Münster, Germany
[2]Clinical Laboratory, University Clinic Münster, Münster, Germany
[3]Department of Neurosurgery, University Hospital Münster, Germany

Correspondence: koenigs@uni-muenster.de

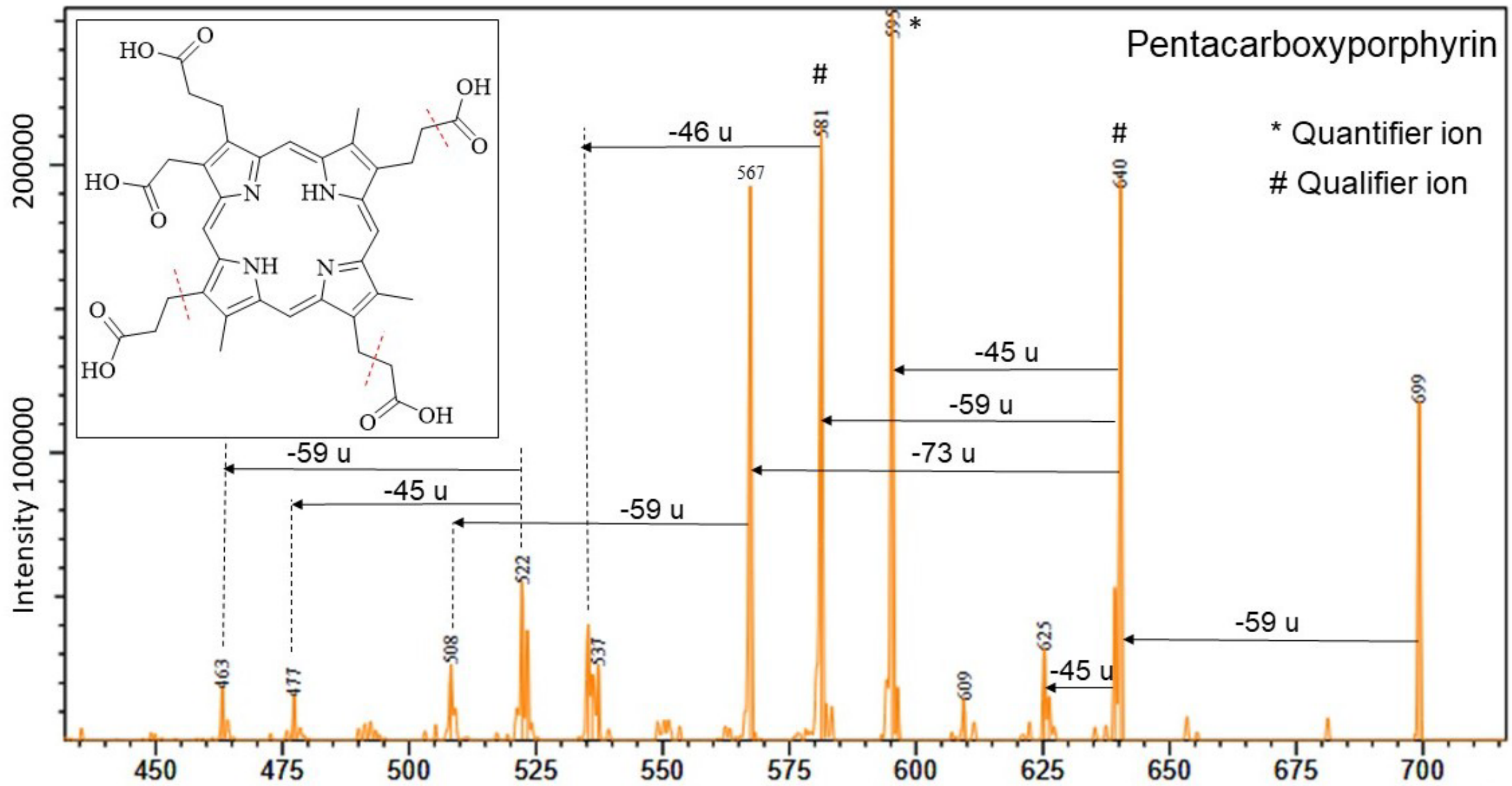

Figure S1: Exemplary MS/MS spectrum obtained for Penta using Shimadzu LCMS-8050. X-axis: *m/z*, y-axis: intensity / counts.

Table S1: Standard deviation of the average peak areas of the ClinTest porphyrin analytes to determine the reproducibility of the purification procedure.

Analyte	Standard deviation / %
Uro	6.84
Hepta	8.90
Hexa	6.40
Penta	6.68
Copro I	5.57
Copro III	6.15

A

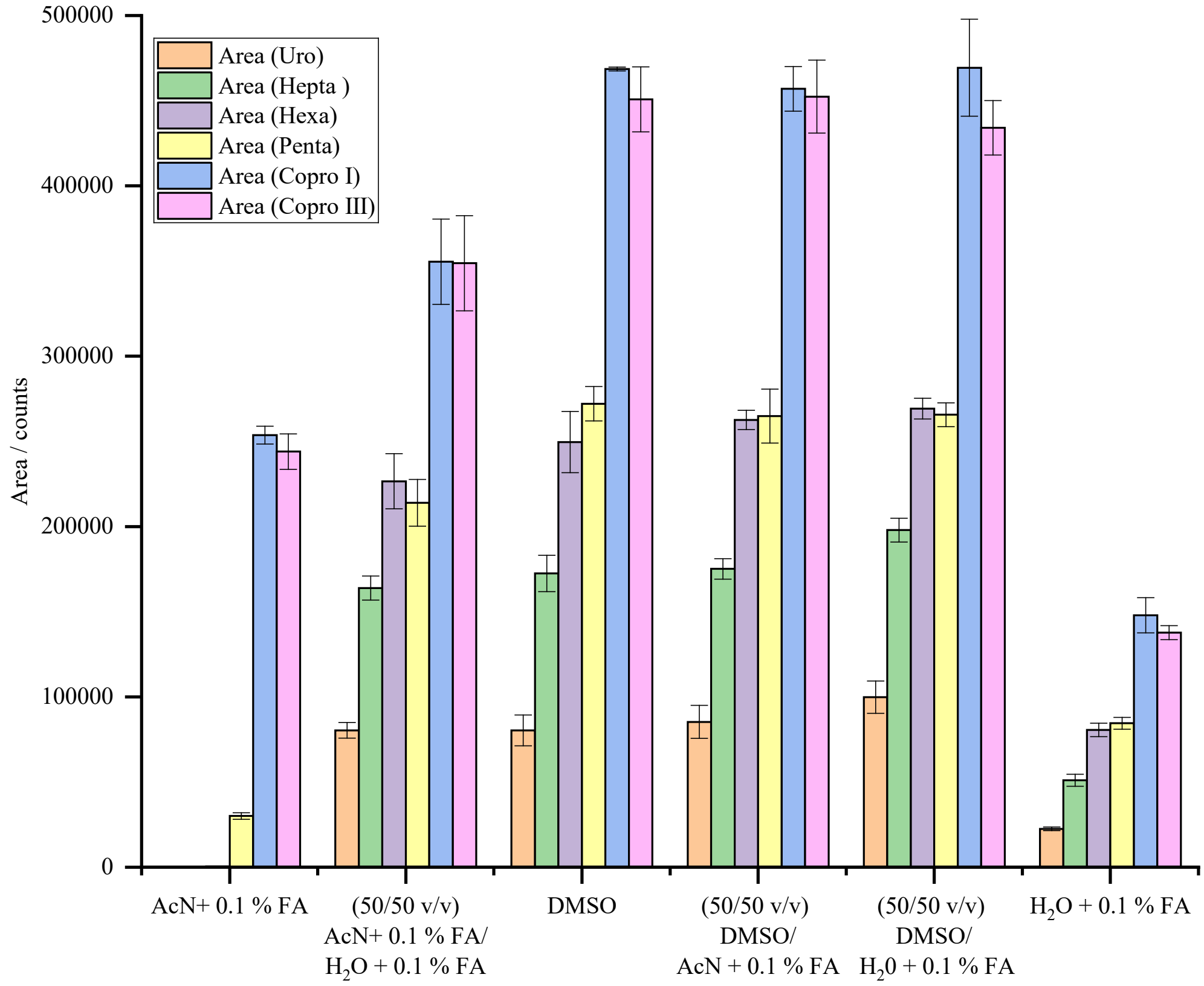

B

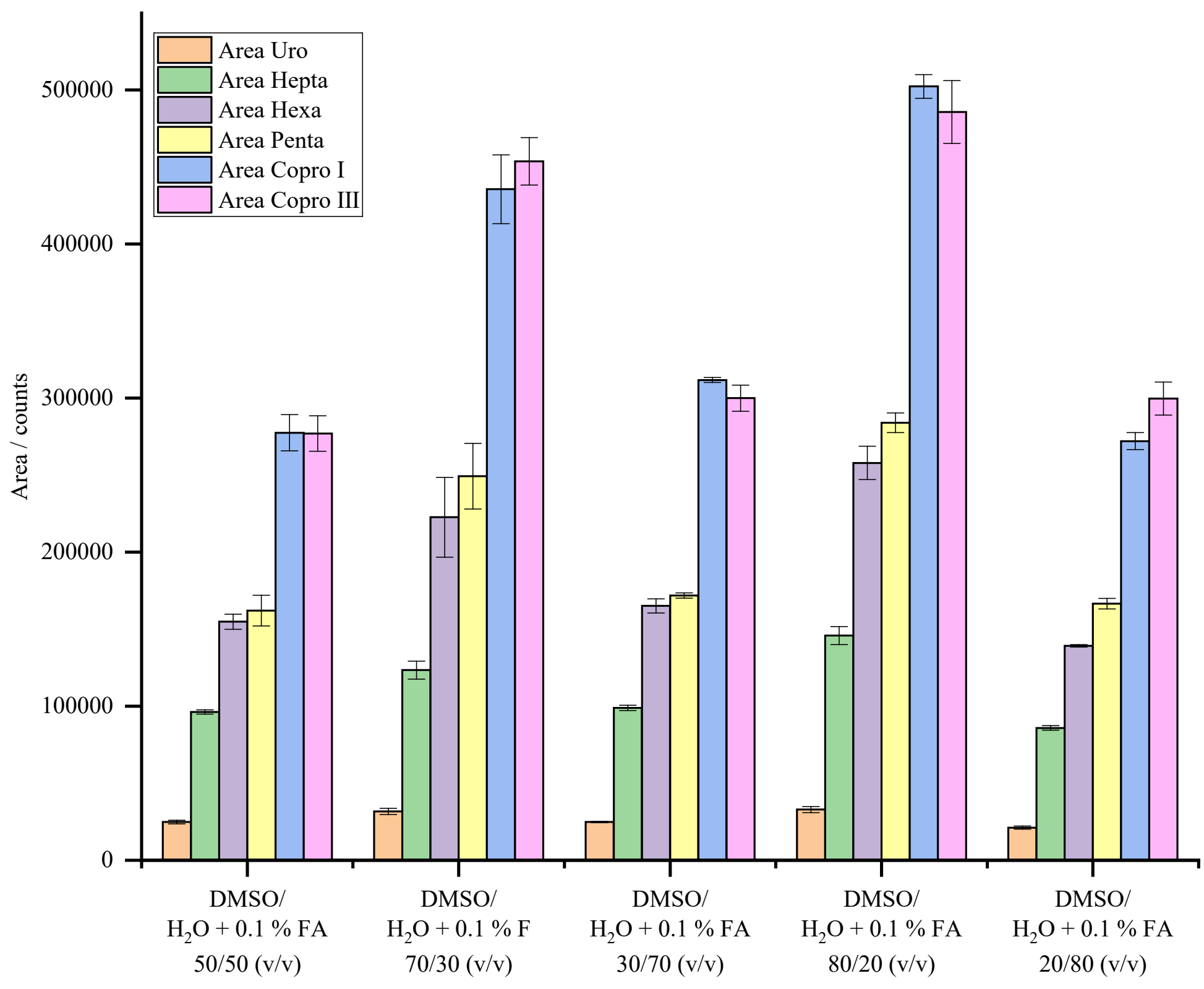

Figure S2: Signal intensities obtained with different injection solvent compositions.

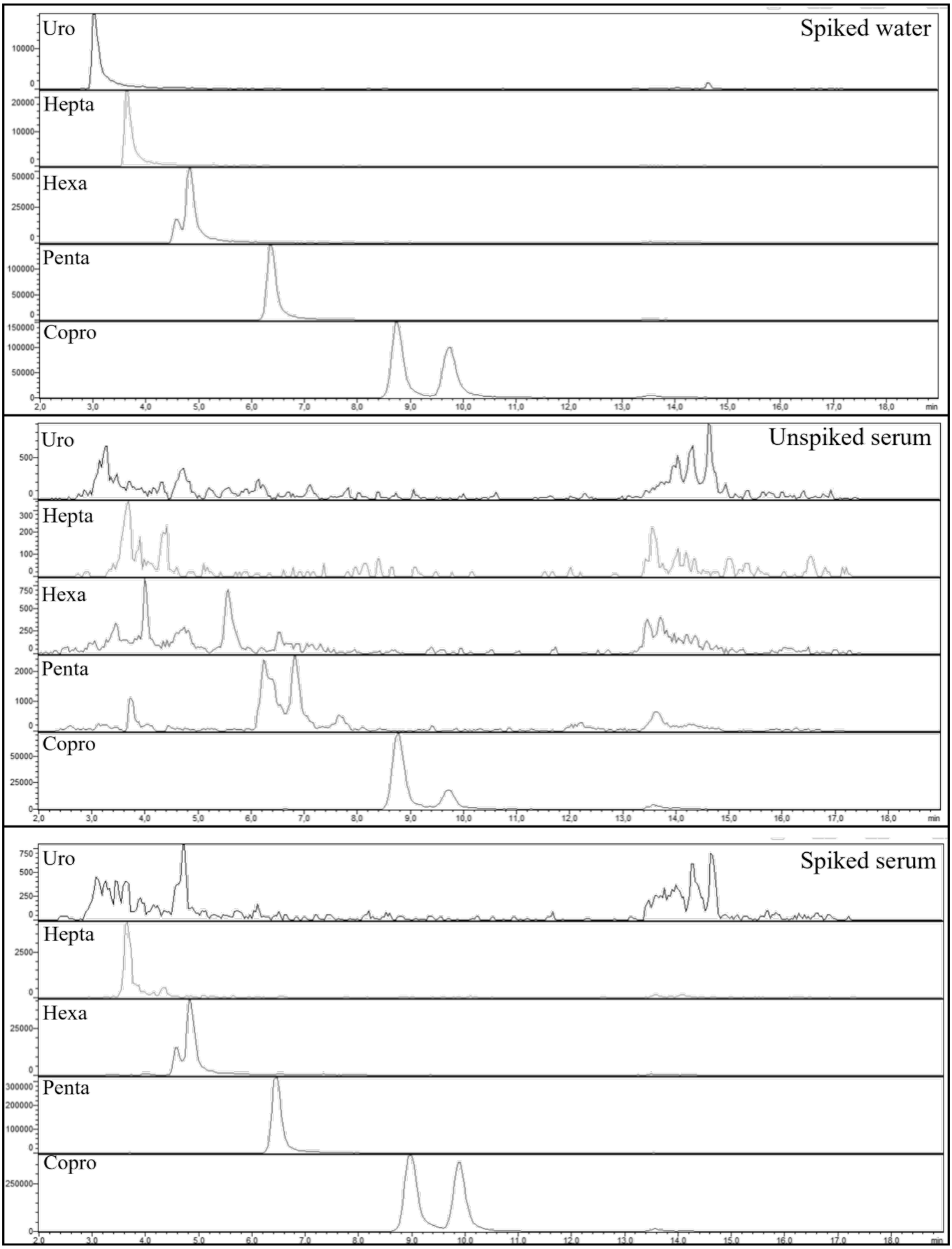

Figure S3: Total ion chromatograms illustrating matrix effects in LC-MS analysis of the porphyrins. Preparation of 500 µl serum and controls. Spike: 0.1 pmol/µl of each porphyrin.

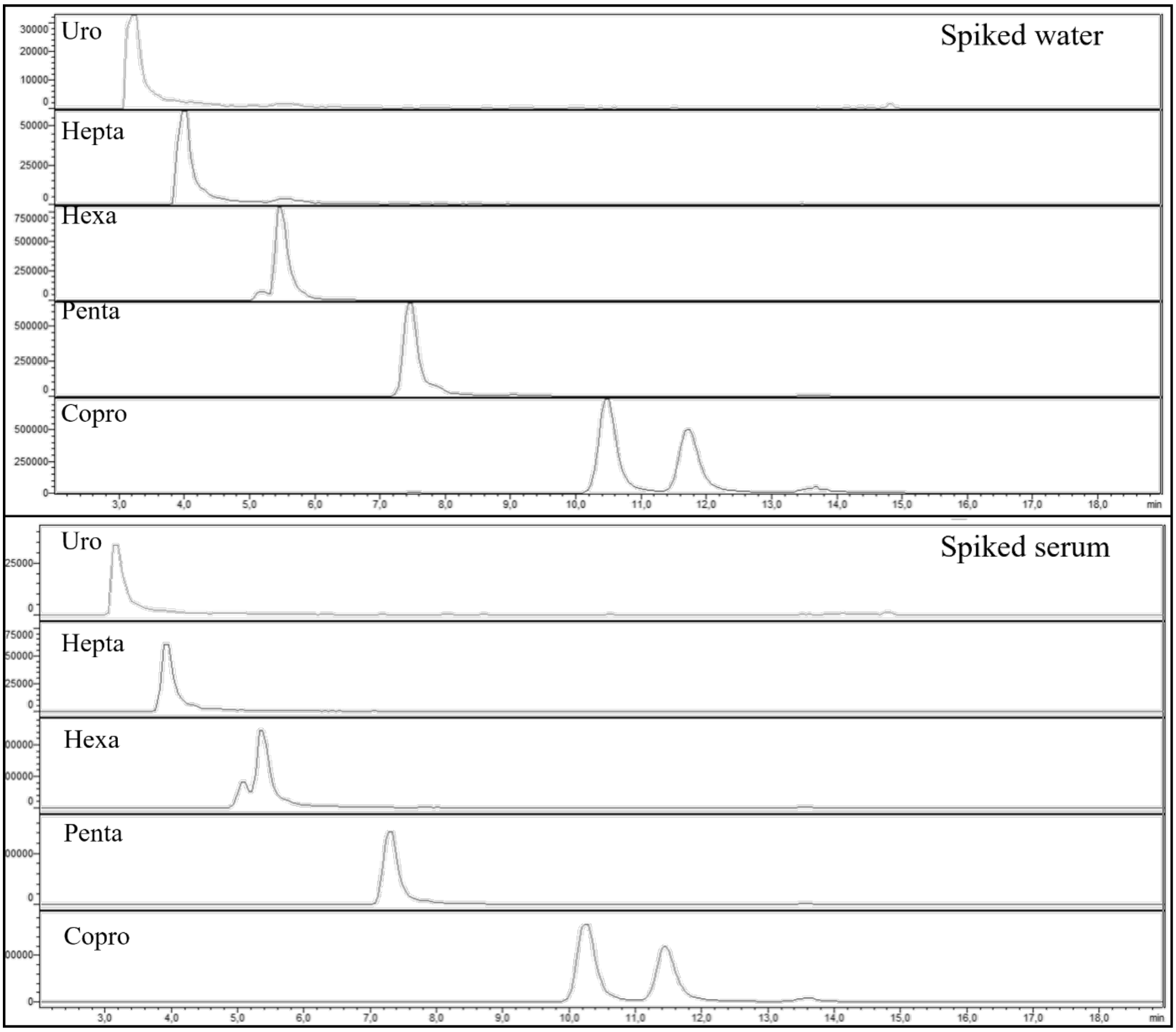

Figure S4: Total ion chromatograms illustrating matrix effects in LC-MS analysis of the porphyrins. Preparation of 100 µl serum and controls. Spike: 0.1 pmol/µl of each porphyrin.

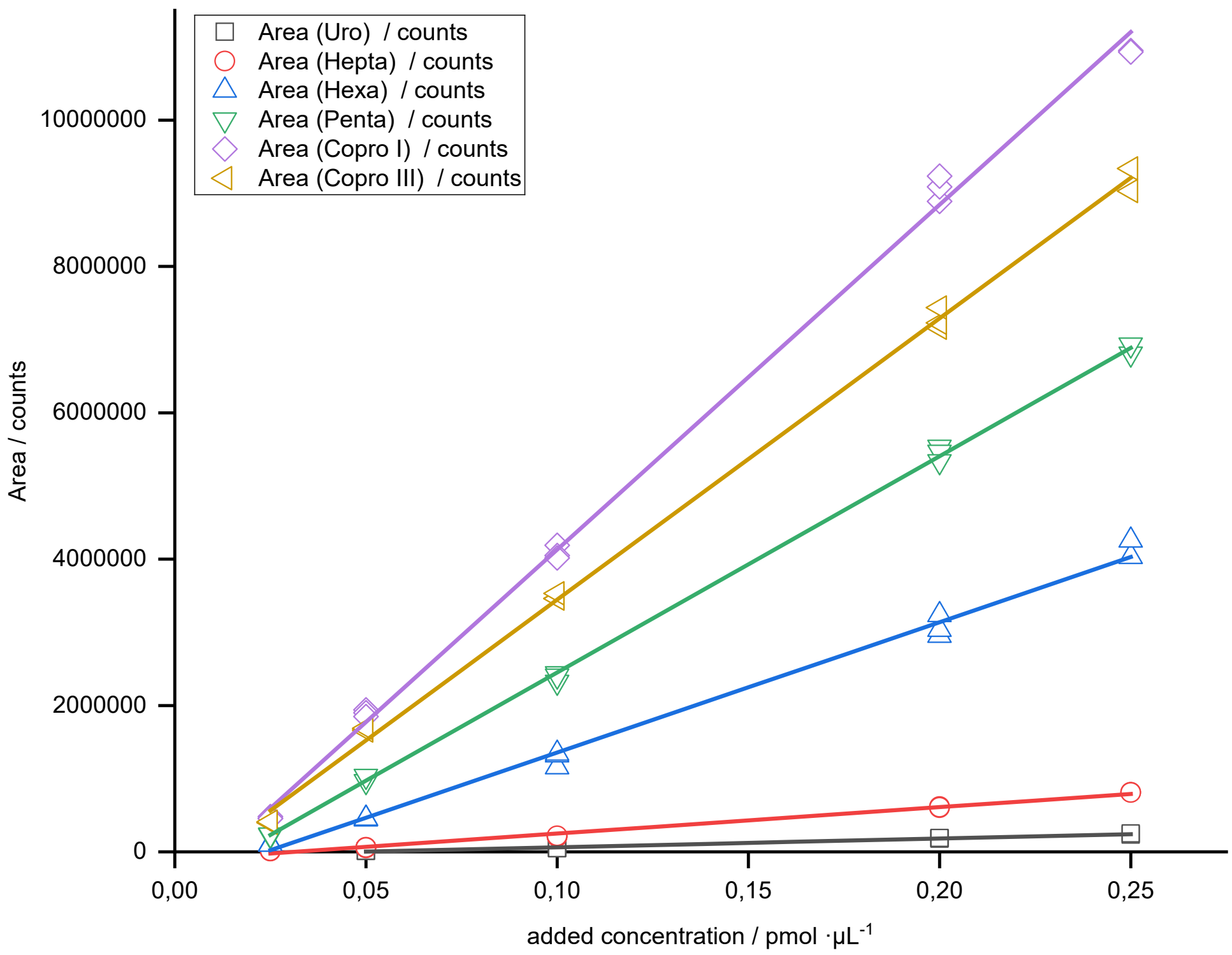

Figure S5: Calibration curves for the porphyrins obtained by standard addition. Data points were acquired by spiking 100 µl serum with varying amounts of the ClinTest standard (5, 10, 20, 40, 50 µl) with subsequent aeSPE and LC-MS. The peak area was obtained from the quantifier ions. R^2 were: Uro 0.990, Hepta 0.993, Hexa 0.995, Penta 0.999, Copro I/III 0.998.

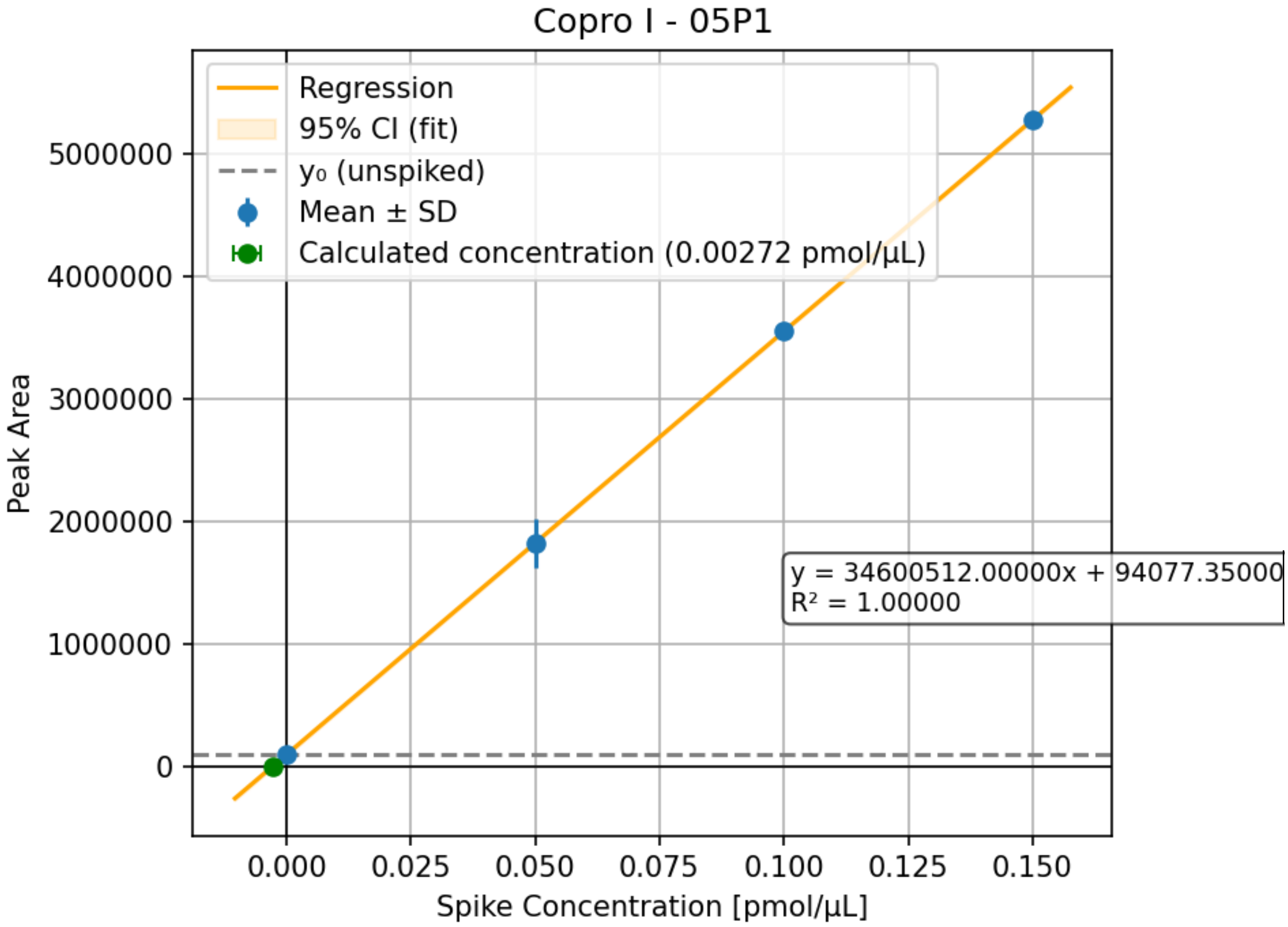

Figure S6: Exemplary output plot from the Python tool

A

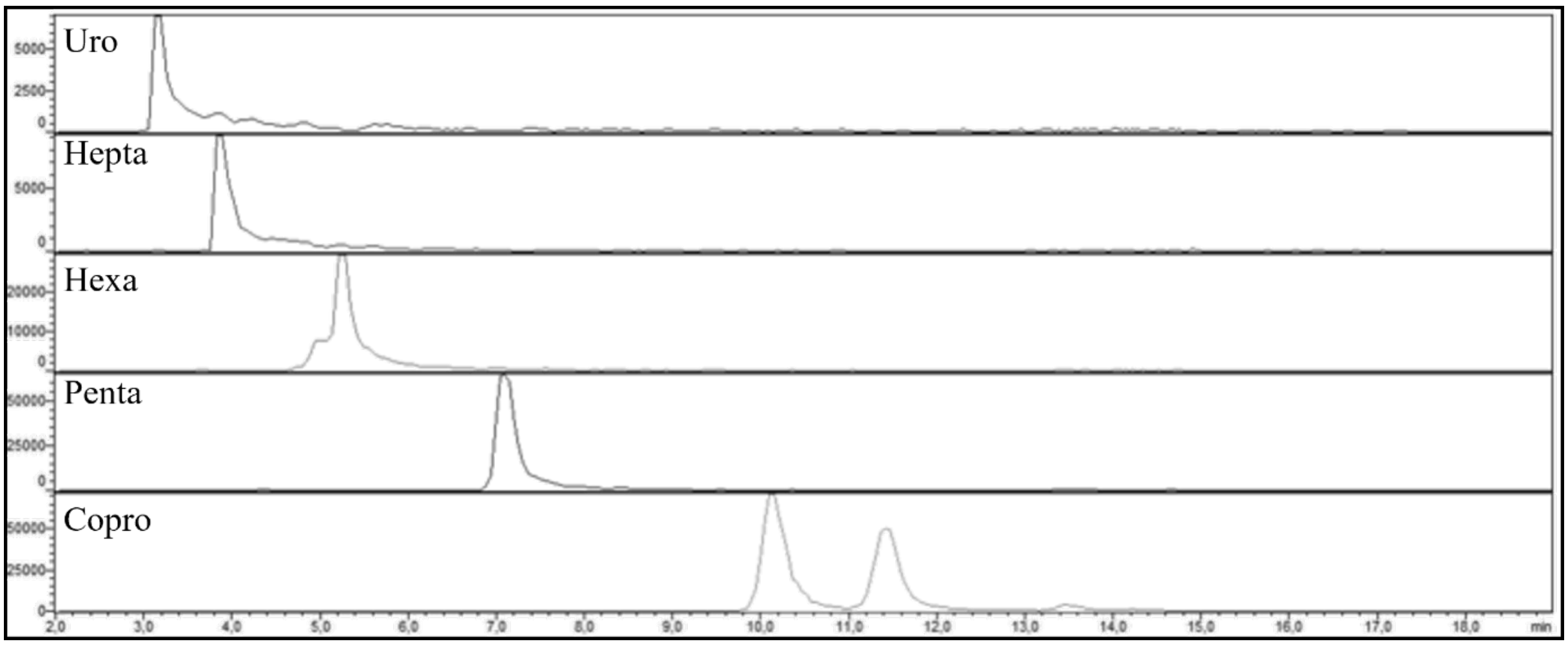

B

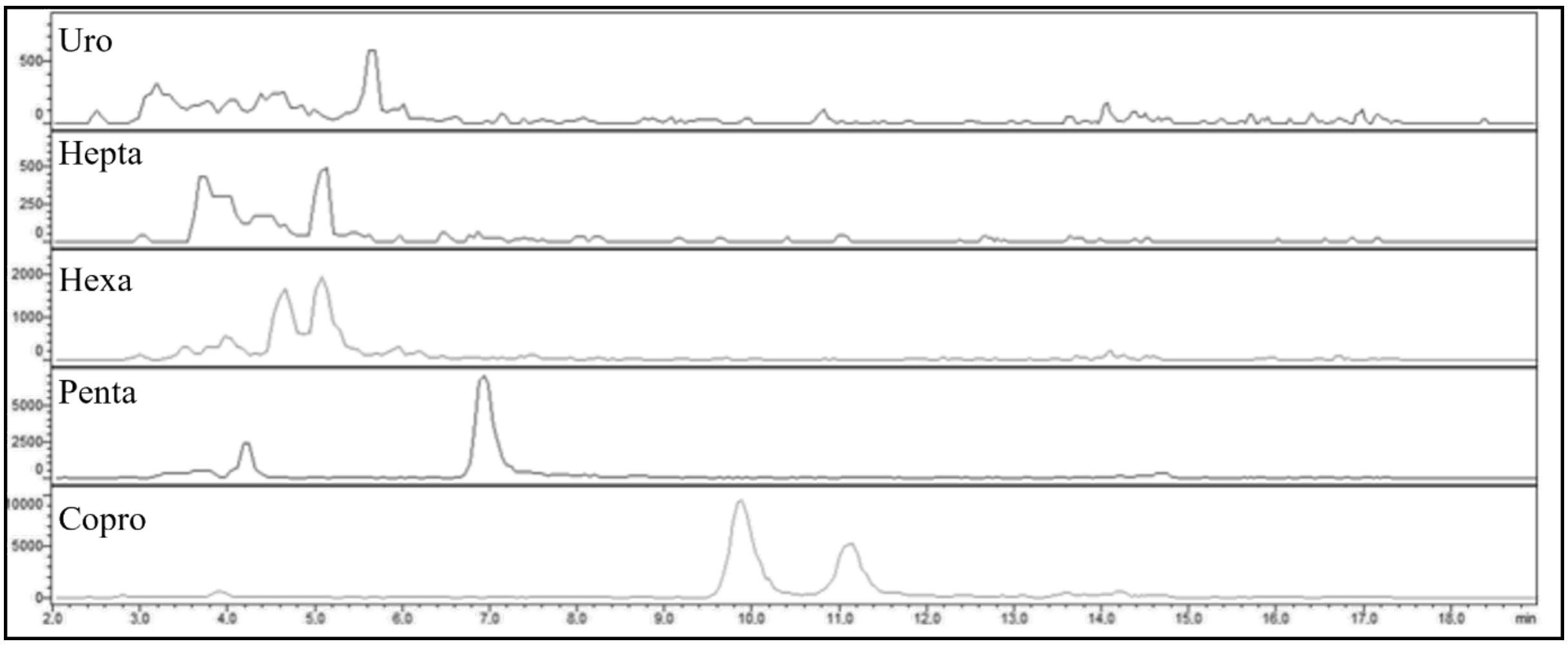

Figure S7: Chromatograms of the porphyrins after LLE with ethyl acetate from spiked water (A) and serum (B). Spikes: 0.1 pmol/µl in the injection solvent.

Supplementary Information for the Python tool

The core functionality of the Python code is based on ordinary least squares (OLS) regression applied to the automatically sorted data from replicate measurements at each spike level. For each combination of sample and analyte, the mean signal at each spike concentration is calculated and a linear regression of peak area against the spiked concentration level is fitted. Exemplary code for OLS regression:

```
# Ordinary Least Squares regression for standard addition
def _ols_simple(x: np.ndarray, y: np.ndarray):
    x_mean = x.mean()
    Sxx = np.sum((x - x_mean) ** 2)
    Sxy = np.sum((x - x_mean) * (y - y.mean()))
    m = Sxy / Sxx
    b = y.mean() - m * x_mean
    return m, b
```

The linear relationship between analytical signal y (counts) and added concentration x (pmol/µl) is expressed as

$$y = m \cdot x + b$$

where m is the slope representing the response per concentration unit and b is the y-intercept corresponding to the signal in the unspiked sample. When the regression is extrapolated to $y = 0$, the intercept with the x-axis x_{int} is obtained. In standard addition, the absolute value x_{int} corresponds to the concentration of the unspiked sample. c_{sample} can thus be calculated by the division of y-intercept b and slope m [1].

$$x_{int} = -\frac{b}{m}$$

$$c_{sample} = \frac{b}{m}$$

Two variables are thus defined. Calculation of the unspiked sample concentration:

```
# ---- Concentration calc ----
        if slope != 0:
            conc_sample = intercept / slope
            conc_abs = abs(conc_sample)
            x_intercept = -intercept / slope
        else:
            conc_sample = conc_abs = x_intercept = float("nan")
```

Additionally, the code calculates uncertainty parameters. Replicate variability of the un-spiked sample consists of the standard deviation (SD) for the triplicate injection ($y_{0,SD}$), which is converted into concentration units by dividing the triplicate SD by the slope m resulting in u_{trip} as an error contribution.

Calculation of the absolute standard deviation of the peak area of the unspiked sample:

$$u_{trip} = \frac{y_{0,SD}}{|m|}$$

This mathematical relationship is expressed as:

```
# ---- Uncertainty from Blank triplicates ----
        if slope != 0 and np.isfinite(y0_sd_peak):
            u_trip_C = y0_sd_peak / abs(slope)
        else:
            u_trip_C = float("nan")
```

The sources of uncertainty of the regression equation consist of the variances of *m* and *b*. Using the law of uncertainty propagation, these are combined to estimate the uncertainty contribution of the regression equation u_{reg}. Mathematically, error propagation uses partial derivatives of c_{sample}, the variance of *b* (*Var(b)*) and the variance of *m* (*Var(m)*) and the covariance (*Cov(m,b)*) in a first order Taylor expansion resulting in a combined variance value for the sample concentration *Var(c)* [2].

Taylor series for the estimation of the error caused by uncertainties of the regression curve:

$$Var(c_{sample}) \approx \left(\frac{\partial C}{\partial m}\right)^2 Var(m) + \left(\frac{\partial C}{\partial b}\right)^2 + Var(b) + \ 2\left(\frac{\partial C}{\partial m}\right)\left(\frac{\partial C}{\partial b}\right) Cov(m,b)$$

The square root of the variance $Var(c_{sample})$ results in the standard uncertainty variable u_{reg} which shows the estimated error contribution by the regression curve.

Mathematical relationship between $Var(c_{sample})$ and u_{reg}.

$$u_{reg} = \sqrt{Var(c_{sample})}$$

To combine both uncertainty contributions u_{reg} and u_{trip} in order to obtain the combined standard uncertainty u_{comb}, the square root of the squared sum of both parameters is calculated.

Calculation of the combined uncertainty of the calculated sample concentration

$$u_{comb} = \sqrt{u_{reg}^2 + u_{trip}^2}$$

In Python, error propagation was expressed as:

```
# ---- Uncertainty from Regression parameters ----
        if slope != 0 and np.isfinite(Var_m) and np.isfinite(Var_b):
            # partials
            dCdM = -intercept / (slope**2)  # -b/m^2  = -(conc_sample)/m
            dCdB = 1.0 / slope              #  1/m
            # variance propagation
            Var_C_reg = (
                (dCdM**2) * Var_m +
                (dCdB**2) * Var_b +
                2.0 * dCdM * dCdB * Cov_mb
            )
            # numeric glitches -> avoid negative due to rounding
            Var_C_reg = Var_C_reg if Var_C_reg > 0 else 0.0
            u_reg_C = np.sqrt(Var_C_reg)
        else:
            u_reg_C = float("nan")

        # ---- Combined uncertainty ----
        if np.isfinite(u_trip_C) and np.isfinite(u_reg_C):
            u_comb_C = np.sqrt(u_trip_C**2 + u_reg_C**2)
        elif np.isfinite(u_trip_C):
            u_comb_C = u_trip_C
        elif np.isfinite(u_reg_C):
            u_comb_C = u_reg_C
        else:
            u_comb_C = float("nan")
```

For full functionality of the code for the evaluation of the standard addition procedure, linearity of the analytical signal with respect to the total analyte concentration is required. The input .csv file must contain columns with sample name, analyte name, spike level and the respective peak area with two or more spike levels and replicates for each sample and analyte. Example:

	A	B	C	D
1	Sample Name	Analyte	Area	Spike Concentration [pmol/µL]
2	05P1	Copro I	102078	0
3	05P1	Copro I	90604	0
4	05P1	Copro I	1681737	0.05
5	05P1	Copro I	1959860	0.05
6	05P1	Copro I	3541554	0.1
7	05P1	Copro I	3566339	0.1

Exemplary output file:

Analyte	Sample	Calculated Concentration [pmol/µL]	absolute standard deviation y-intercept (Peak Area)	relative standard deviation y-intercept [%]	absolute standard deviation calculated sample concentration based on regression (delta b/m) [pmol/µL]	absolute deviation regression [pmol/µL]	combined absolute standard deviation [pmol/µL]	combined relative standard deviation [%]	Regression Equation	R^2
**Copro I**	05P1	0.00272	8113.343	8.421485	0.000234	7.34E-05	0.000246	9.036269	y = 3.46005e+07x + 94077.3	1.00000
**Copro III**	05P1	0.00914	22735.6	9.120893	0.000846	0.000441	0.000954	10.43485	y = 2.68789e+07x + 245685	0.99996

The determined concentration in column 3 corresponds to the concentration of the analyte in the LC-MS injection solution, in our case to the 40 µl obtained following aeSPE. The values need to be transformed to the concentration in serum by considering the starting volume of the experiment (here: 100 µl serum).

1. Rodríguez, L.C.; Campaña, A.M.G.; Barrero, F.A.; Linares, C.J.; Ceba, M.R. Validation of an Analytical Instrumental Method by Standard Addition Methodology. J AOAC Int 1995, *78*, 471–476, doi:10.1093/jaoac/78.2.471.
2. Soch, J. The Book of Statistical Proofs, 2025. Available online: https://statproofbook.github.io/ (accessed on 21 October 2025).

Recommended folder structure of the Python project in Visual Studio Code

```
project_root/
│
├── analyzer.py                # file with the code
├── dummy file.csv   # input CSV file of the chromatographic data
│
├── results/                   # created automatically by the script
│   ├── HPLC_Results.xlsx          # main Excel output (generated by the script)
│   └── plots/                     # subfolder for plots (generated by the script)
│       ├── Sample1_Analyte1.png
│       ├── Sample1_Analyte2.png
│       └── ...
```

It is very important that for calculation of each new experiment a separate file folder is generated, which contains the input file (filename.csv) and the Python script (HPLC_analyzer_porphyrins_standard_addition.py). The results file will be created in this folder. The folder needs to be opened in VSC and then the script can be run.

Installation information

Visual Studio Code (VSC) was used as Python platform. https://code.visualstudio.com/

The following Python packages require installation: numpy, pandas, matplot, xlsxwriter and scipy. In our experience, this can simply be done with the command

pip install numpy and pandas

exemplarily, following installation of VSC. However, we did encounter difficulties with new Python installations both in network and single environments with the pip command not working. For a fix, we recommend to:

1. Install VSC
2. Install Python 3.14 and tell Python Interpreter in VSC where to find it.
3. Generate a VSC network environment (.venv folder) in which Python packages can be obtained directly.
4. Check bottom line in VSC, if Python and the environment are recognized (here: pippython.venv)

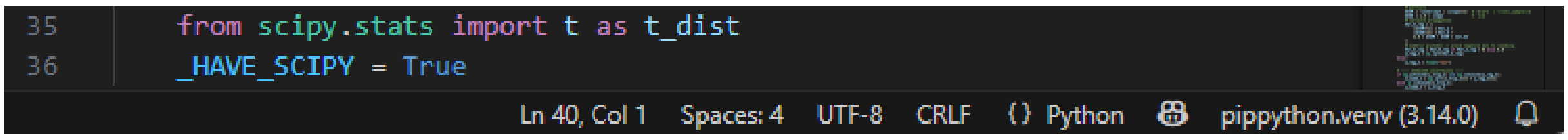

For additional information, see:

Torniainen, J.; Afara, I.O.; Prakash, M.; Sarin, J.K.; Stenroth, L.; Töyräs, J. Open-source python module for automated preprocessing of near infrared spectroscopic data. Analytica Chimica Acta 2020, 1108, 1-9, doi.org/10.1016/j.aca.2020.02.030
